# Acupuncture treatment on attention deficit hyperactivity disorder: A protocol for systematic review and meta-analysis

**DOI:** 10.1097/MD.0000000000027033

**Published:** 2021-08-27

**Authors:** Liwei Xing, Zeqin Ren, Xianwu Yue, Hongxi Chen, Chunlin Xia, Fan Liu, Qinzuo Dong, Kenan Wu, Rong Zhao

**Affiliations:** a Yunnan University of Chinese Medicine, 88 Baita Road, Panlong District, Kunming city, Yunnan Province, China; b First Affiliated Hospital of Dali University, No. 32, Jiashibo Avenue, Dali Bai Autonomous Prefecture, Dali City, Yunnan Province, China.

**Keywords:** acupuncture, attention deficit hyperactivity disorder, comprehensive treatment, meta-analysis, protocol

## Abstract

**Background:**

Attention Deficit Hyperactivity Disorder (ADHD) is the most common behavioral disorder in childhood. Acupuncture treatment of ADHD has formed a relatively systematic theoretical and clinical treatment system which achieved satisfactory results. However, there has been no systematic evaluation of its effectiveness and safety. The purpose of this study was to evaluate the efficacy and safety of acupuncture in the treatment of ADHD.

**Methods:**

A systematic search of literature will be conducted in PubMed, Cochrane Library, the Web of Science, Excerpt Medica Database, Chinese Biomedical Literature Database, VIP, Wanfang database, China National Knowledge Infrastructure database for articles published up to September, 2019. The searching terms include “attention deficit”, “hyperactivity”, “mild brain dysfunction”, “acupuncture”, “electroacupuncture”. The search is limited to studies published in Chinese and English. Two reviewers will extract and evaluate the information independently. Cochrane Collaboration tool and Jadad scale will be used to evaluate the quality of the studies. Review Manager Version 5.3 (Cochrane Collaboration's software) will be used to carry out the meta-analysis.

**Results:**

High-quality synthesis and/or descriptive analysis of current evidence will be provided from effective rate, total score of traditional Chinese medicines syndromes, conners child hyperactivity-diagnosis rating scale, conners index of hyperactivity, the recurrence rate, and adverse events.

**Conclusion:**

This study will provide the evidence of whether acupuncture is an effective and safe intervention to ADHD.

**INPLASY registration number:**

INPLASY202140022

## 1 Introduction

Attention Deficit Hyperactivity Disorder (ADHD) is the most common behavioral disorder in childhood. Its core symptoms are inconsistency, hyperactivity and age-inappropriate impulsive behavior. Learning disabilities, other psychological and pathological manifestations have a profound impact on children's academic performance, quality of life, and so on.^[1]^ The prevalence rate among school-age children is 3% to 5%, and the male-to-female incidence ratio is (4–9):1.^[2]^ Treatment for ADHD includes psychological support, behavior modification, and medication. Among them, central stimulants such as Ritalin and haloperidol are used. However, long-term use of central stimulants may cause adverse reactions, such as insomnia, headache, and loss of appetite. Therefore, scholars at home and abroad have been searching for more effective and less side effects alternative medical therapies to treat diseases, including acupuncture, even if there is insufficient evidence-based medical evidence for its clinical efficacy.^[3]^

Acupuncture treatment of ADHD started in the early 1980s.^[4]^ In the past 30 years, acupuncture treatment of ADHD has formed a more systematic theory and clinical treatment system, which has achieved satisfactory curative effects, and has certain advantages and development potential.^[5]^ Acupuncture is a method to treat ADHD, and its clinical efficacy has been gradually recognized and promoted in western countries. Researchers have tried to treat ADHD patients with acupuncture and achieved remarkable clinical effects.^[6,7]^ Cochrane Collaboration^[8]^ and Lee et al^[9]^ conducted a systematic review and meta-analysis of acupuncture treatment for ADHD in 2010 respectively. The former performed a descriptive analysis of the included studies and the meta-analysis of only 3 included studies. The results showed that acupuncture cannot be considered symptomatic to treat ADHD. Ni et al^[10]^ conducted a new meta-analysis on acupuncture for ADHD in 2015, including 13 literatures. Although this was an increase compared with previous studies, there were still deficiencies in terms of literature quality, scientific design of the trial, lack of unified evaluation indicators and other aspects. There have been no controlled studies of acupuncture versus sham acupuncture. In recent years, people's understanding of ADHD has been gradually improved, and the incidence of ADHD in China has been increasing year by year. In addition, relevant clinical trials have been published, and the international application of acupuncture is becoming more and more widespread. It is necessary to update and supplement the systematic review of ADHD. Therefore we decided to conduct an evidence-based review to evaluate the efficacy and safety of acupuncture in the treatment of ADHD.

## 2 Methods

The study aims to evaluate the effectiveness and safety of acupuncture in the treatment of ADHD. We will use the preferred reporting items for systematic reviews and meta-analysis statement to guide our systematic evaluation report.^[11]^ And the review was registered in the international platform of registered systematic review and meta-analysis protocols (INPLASY) database (INPLASY202140022).

### 2.1 Data sources and retrieval strategy

Comprehensive retrieval databases include the following databases: Cochrane Library, PubMed, Excerpt Medica Database; Chinese Academic Journal Full-text Database, Chinese Biomedical Literature Database, Weipu Chinese Science and Technology Journal Full-text Database (VIP), Wanfang Data Knowledge Service Platform (Wanfang), the time limitation is from the construction of the library to September 2019. We will develop a corresponding search strategy under the guidance of the cochrane search guide. The relevant meeting records, trial registers, and a reference list of publications identified will also be searched for further trials. The following group terms will be used for searching (acupuncture or acupuncture therapy or acupuncture, ear or acupuncture points or acupuncture analgesia) and (ADHD or attention deficit hyperactivity disorder or attention deficit disorder with hyperactivity or ADDH).

The database will be searched by combining subject words and random words. Taking PubMed retrieval as an example, the retrieval strategy is shown in Table 1. This search strategy will be modified and used for the other databases.

**Table 1:** Retrieval strategy of PubMed.

Number	Search term
#1	“Acupuncture” [MeSH] or “Acupuncture Therapy ” [Title/Abstract] or “Acupuncture, Ear” [Title/Abstract] or “Acupuncture Points” [Title/Abstract] or “Acupuncture Analgesia” [Title/Abstract]
#2	“ADHD” [Title/Abstract] or “attention deficit hyperactivity disorder” [Title/Abstract] or “Attention Deficit Disorder with Hyperactivity” [Title/Abstract] or “ADDH” [Title/Abstract]
#3	“Randomized controlled trial” [Title/Abstract] or “Controlled clinical trial ” [Title/Abstract]
#4	#1 and #2 and #3

MeSH = medical subject headings.

### 2.2 Eligibility criteria

#### 2.2.1 Types of participants

ADHD patients with clear diagnostic criteria.

#### 2.2.2 Types of interventions and comparators

The intervention group received traditional acupuncture or other treatment methods, while the control group received sham acupuncture or placebo drugs or proven targeted therapy (such as western medicine, behavioral therapy, etc).

#### 2.2.3 Types of outcomes

The primary prognostic indicators are effectiveness, and the secondary prognostic indicators are symptoms or symptom scores (Connors Child Behavior Scale, Hyperactivity Index, etc), adverse reactions, etc.

#### 2.2.4 Types of studies

The selected articles should be randomized controlled trials and clinical controlled trials.

### 2.3 Study selection and data extraction

**Figure 1:**
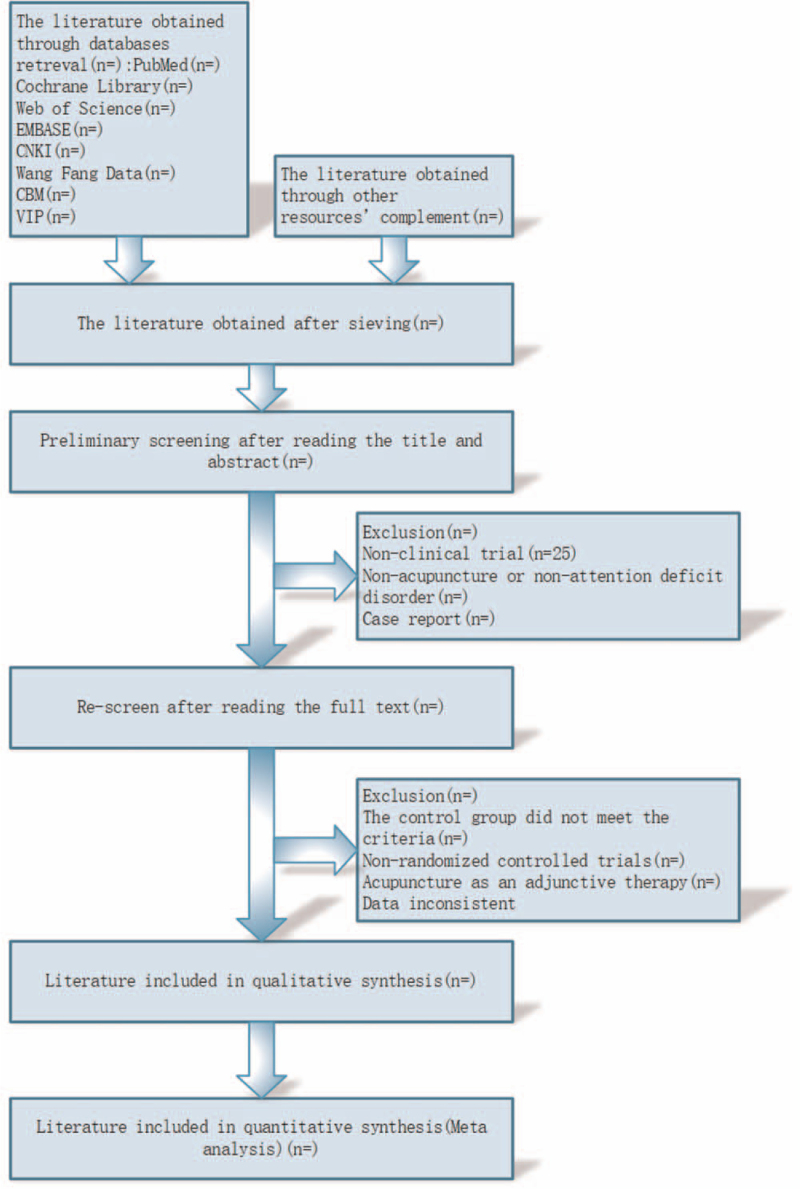
PRISMA flow chart. PRISMA = preferred reporting items for systematic reviews and meta-analysis.

Endnote X9.0 (Clarivate Analytics’ software) will be used to manage the retrieved studies. Preliminary screening involves reading titles and abstracts to eliminate duplicates and ineligible studies. Re-screening involves reading the full text and selecting studies based on inclusion and exclusion criteria. Differences between the 2 reviewers will be resolved through discussion, with a third reviewer consulted if necessary. The preferred reporting items for systematic reviews and meta-analysis flow chart was shown in Figure 1.^[12]^

Two reviewers (Zeqin Ren and Liwei Xing) will independently screen the titles, abstracts, and keywords of the retrieved studies and further assess the full texts. Excluded studies were recorded with explanations. Two reviewers will independently extract the data and complete the predefined data extraction form. General information, participants, methods, interventions, outcomes, results, adverse events, conflicts of interest, ethical approval, and other information will be extracted. Disagreements will be solved by discussion between the 2 reviewers and arbitrated by the third reviewer (Xianwu Yue) when necessary. Authors of the studies will be contacted for clarification and missing data.

### 2.4 Risk of bias assessment

The Cochrane risk bias assessment tool^[13]^ will be used to evaluate the methodological quality of the included literature: including random methods, allocation hiding methods, blind methods, baselines, intentionality analysis, number of missing follow-ups, and reasons for withdrawal, and the quality of the literature was classified as A (Less bias), B (medium bias), C (high bias).^[14]^ The literature included in the evaluation will be independently evaluated by 2 reviewers (Hongxi Chen and Chunlin Xia) in the research team, and in case of disagreement, a third reviewer (Fan Liu) will participate in the discussion and settlement.

### 2.5 Statistical analysis

#### 2.5.1 Heterogeneity test and meta-analysis

RevMan 5.3 software provided by Cochrane Collaboration Network will be used for meta-analysis.^[15]^ Outcome indicators are count data. Relative risk will be used as the effect indicator, and 95% confidence interval will be used for statistical analysis. I^2^ statistics will be selected for heterogeneity testing. The low, medium, and high degrees of heterogeneity will be expressed by I^2^ statistics of 25%, 50%, and 75%, respectively. If there is statistical homogeneity (*P* > .1, I^2^ < 50%) between the results of each study, a fixed effects model will be used for analysis; if there is statistical heterogeneity between the results (*P* < .1, I^2^ > 50%), the source of heterogeneity will be analyzed; if there is statistical heterogeneity between the 2 study groups without clinical heterogeneity or the difference is not statistically significant, a random effect model (random effects model will be used, otherwise the descriptive analysis.^[16]^

#### 2.5.2 Sensitivity analysis

Choose different statistical models (fixed effects model/random effects mode) for meta-analysis of outcome indicators, and evaluate the combined effect. If there is no substantial change in the results before and after the sensitivity analysis, the meta-analysis results are more credible; if the sensitivity analysis results are significantly different, it indicates that the meta-analysis results are less robust, that is, there are potential other factors related to the effect of the intervention, Therefore, the interpretation of the results should be more conservative.

#### 2.5.3 Assessment of publication biases

The funnel chart drawn by RevMan 5.3 software will be used to detect publication bias.

#### 2.5.4 Subgroup analysis

Subgroup analysis will be performed based on the results of data synthesis if the heterogeneity is high. The following subgroup analyses will be considered: intervention methods (type, time) and measures used in clinical trials.

#### 2.5.5 Grading the quality of evidence

The GRADE profiler 3.2 (Evidence Prime's software) will be used for analysis. The quality of evidence will be divided into 4 levels: high, medium, low, and very low.^[17]^

## 3 Discussion

Acupuncture is a traditional treatment method, and its clinical efficacy has gradually been recognized and promoted on ADHD. Acupuncture has the function of calming the mind, regulating the viscera, filling the marrow, promoting the intellectual development and so on. In recent years, with the increasing awareness of ADHD from all walks of life, more and more clinical trials have been conducted on the application of acupuncture in ADHD. It is necessary to update and supplement the systematic review of ADHD. We hope the results of this study may provide evidence regarding acupuncture treatment on ADHD.

## Acknowledgments

We acknowledged these fund projects which contributed towards the article.

## Author contributions

**Conceptualization:** Rong Zhao.

**Data curation:** Xianwu Yue, Hongxi Chen, Chunlin Xia.

**Investigation:** Liwei Xing, Zeqin Ren.

**Methodology:** Fan Liu, Kenan Wu.

**Supervision:** Fan Liu, Kenan Wu.

**Visualization:** Qinzuo Dong.

**Writing – original draft:** Liwei Xing, Zeqin Ren.

**Writing – review & editing:** Rong Zhao.
